# The Relationship Between Hematological Findings and Coronary Artery Aneurysm in Kawasaki Disease

**DOI:** 10.4274/tjh.2013.0241

**Published:** 2014-06-10

**Authors:** Burçin Beken, Şule Ünal, Mualla Çetin, Fatma Gümrük

**Affiliations:** 1 Hacettepe University Faculty of Medicine, Department of Pediatric Hematology, Ankara, Turkey

**Keywords:** Thrombocytopenia, Kawasaki disease, Aneurysm

## TO THE EDITOR

Kawasaki disease (KD) is a self-limited systemic vasculitis that occurs predominantly in children. It is diagnosed by using a clinical case definition that requires fever (≥5 days) together with 4 of 5 principal clinical criteria including changes in extremities, polymorphous exanthem, bilateral bulbar conjunctival injection without exudate, changes in lips and oral cavity, and cervical lymphadenopathy [1,2,3]. Patients with KD are known to have a number of common hematological abnormalities including leukocytosis and thrombocytosis. Thrombocytosis is usually seen during the acute phase of the disease, whereas thrombocytopenia is seen rarely in the acute stage and the presence of thrombocytopenia has been reported to be a risk factor for coronary aneurysm development [4,5,6]. 

Thirty-seven patients who were diagnosed with KD between June 2007 and January 2013 in the Hacettepe University İhsan Doğramacı Children’s Hospital were retrospectively evaluated for the hematological findings at presentation, and the hematological parameters were further analyzed for the predictivity of coronary aneurysm development. 

Hematological parameters of these patients are shown in Table 1. Of these patients, 18 (48.6%) had thrombocytosis. Seventeen (45.9%) patients had normal thrombocyte levels and 2 patients (5.4%) had thrombocytopenia in the acute phase of the disease. In total, 15 (40%) patients had coronary artery aneurysm at diagnosis, including both of the 2 thrombocytopenic patients. The patients with aneurysm (Group 1) had lower hemoglobin and hematocrit levels and higher WBC counts than the patients without aneurysm (Group 2). Patients with WBC counts above 15.85x109/L were found to have increased coronary artery aneurysm risk of up to 6.6-fold (95% CI: 1.5-29.6), with a sensitivity of 71.4% and a specificity of 27.3%. Hemoglobin levels under 10.6 g/dL at presentation were found to increase the coronary artery aneurysm risk by up to 4.5-fold (95% CI: 1.0-19.4) with a sensitivity of 57.1% and a specificity of 23.8% , while hematocrit levels under 30.2 g/dL increased the coronary artery aneurysm risk 4.2-fold (95% CI: 0.9-19.2) with a sensitivity of 50% and a specificity of 19%.

In the past, thrombocytosis was thought to be essential for the diagnosis of KD, and in the presence of thrombocytopenia, investigation of another diagnosis was recommended. However, in the past few years, this view has changed. Thrombocytopenia can be seen as a rare finding in KD and may be reported to be present in the acute phase of the disease, usually on days 5-12, disappearing within 3-4 days [4]. 

Niwa et al. reported 10 patients with thrombocytopenia among 303 patients with KD [6]. Six of 10 patients with thrombocytopenia (60%) developed aneurysms. In some reports, thrombocytopenia was found to be associated with acetylsalicylic acid [5]. Another suspected theory for the mechanism of thrombocytopenia is immune thrombocytopenic purpura during the course of KD [7,8]. Krowchuk et al. suggested that thrombocytopenia is related to the destruction of thrombocytes by immunoglobulins or non-immune mechanisms [9]. The rate of thrombocytopenia association in KD has been reported as 1%-2% [5]. Turkish patients with KD have been previously reported to develop earlier aneurysm and desquamation [10]; herein, we also report a higher rate (5.4%) of thrombocytopenia association in these patients at presentation, which may also indicate earlier aneurysm development in thrombocytopenic patients. 

In conclusion, thrombocytopenia can also be seen as a presenting feature in KD instead of thrombocytosis. Anemia, leukocytosis, and thrombocytopenia should alert the clinician of severe disease and coronary artery aneurysm development, and closer follow-up with more frequent echocardiographic examinations may be a safer approach in these patients.

## CONFLICT OF INTEREST STATEMENT

The authors of this paper have no conflicts of interest, including specific financial interests, relationships, and/ or affiliations relevant to the subject matter or materials included.

## Figures and Tables

**Table 1:**
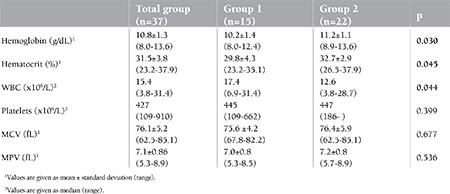
Comparison of hemogram findings of KD patients with (Group 1) or without (Group 2) coronary artery aneurysm.
